# A Critical Review of Pro-Cognitive Drug Targets in Psychosis: Convergence on Myelination and Inflammation

**DOI:** 10.3389/fpsyt.2014.00011

**Published:** 2014-02-04

**Authors:** Rune A. Kroken, Else-Marie Løberg, Tore Drønen, Renate Grüner, Kenneth Hugdahl, Kristiina Kompus, Silje Skrede, Erik Johnsen

**Affiliations:** ^1^Division of Psychiatry, Haukeland University Hospital, Bergen, Norway; ^2^Department of Biological and Medical Psychology, University of Bergen, Bergen, Norway; ^3^Department of Radiology, Haukeland University Hospital, Bergen, Norway; ^4^Department of Physics and Technology, University of Bergen, Bergen, Norway; ^5^NORMENT Center of Excellence, University of Bergen, Bergen, Norway; ^6^Dr. Einar Martens’ Research Group for Biological Psychiatry, Centre for Medical Genetics and Molecular Medicine, Haukeland University Hospital, Bergen, Norway; ^7^Department of Clinical Science, K.G. Jebsen Centre for Psychosis Research, University of Bergen, Bergen, Norway; ^8^Department of Clinical Medicine, University of Bergen, Bergen, Norway

**Keywords:** schizophrenia, cognition, glutamate, myelin, inflammation, immunology, connectivity, neuroimaging

## Abstract

Antipsychotic drugs have thus far focused on dopaminergic antagonism at the D2 receptors, as counteracting the hyperdopaminergia in nigrostriatal and mesolimbic projections has been considered mandatory for the antipsychotic action of the drugs. Current drugs effectively target the positive symptoms of psychosis such as hallucinations and delusions in the majority of patients, whereas effect sizes are smaller for negative symptoms and cognitive dysfunctions. With the understanding that neurocognitive dysfunction associated with schizophrenia have a greater impact on functional outcome than the positive symptoms, the focus in pharmacotherapy for schizophrenia has shifted to the potential effect of future drugs on cognitive enhancement. A major obstacle is, however, that the biological underpinnings of cognitive dysfunction remain largely unknown. With the availability of increasingly sophisticated techniques in molecular biology and brain imaging, this situation is about to change with major advances being made in identifying the neuronal substrates underlying schizophrenia, and putative pro-cognitive drug targets may be revealed. In relation to cognitive effects, this review focuses on evidence from basic neuroscience and clinical studies, taking two separate perspectives. One perspective is the identification of previously under-recognized treatment targets for existing antipsychotic drugs, including myelination and mediators of inflammation. A second perspective is the development of new drugs or novel treatment targets for well-known drugs, which act on recently discovered treatment targets for cognitive enhancement, and which may complement the existing drugs. This might pave the way for personalized treatment regimens for patients with schizophrenia aimed at improved functional outcome. The review also aims at identifying major current constraints for pro-cognitive drug development for patients with schizophrenia.

## Introduction

Schizophrenia is a severe mental disorder with typical onset in the late teens or early adulthood and a chronically relapsing remitting course in the majority of cases (1). Schizophrenia remains a leading cause of years lived with disability (2), and active psychosis has been ranked among the most disabling disorders by severity in the general population (3).

The clinical picture of schizophrenia is heterogeneous, but in the vast majority of patients, cognitive dysfunctions are present with adverse impact on daily functioning (1). Antipsychotic drugs have for six decades been a cornerstone in the treatment of the disorder but a fundamental limitation is their small effect sizes in the cognitive symptom domain (4) as the drugs are primarily effective against the positive symptoms of psychosis including hallucinations and delusions (4). Since the serendipitous discovery of chlorpromazine’s antipsychotic properties, dopaminergic antagonism has served as a template for all subsequent antipsychotics through counteracting subcortical hyperdopaminergia (5). The drugs following chlorpromazine are usually classified as typical or first generation antipsychotics (FGAs), whereas clozapine much later became the prototype for the atypical or second-generation antipsychotic (SGA) drugs that were developed in the 1990s onward (4, 6). Only modest differences in antipsychotic efficacy have been found among FGAs and SGAs (7), but the SGAs seem to display a broader therapeutic repertoire including also pro-cognitive properties, although of a small magnitude (8). Pharmacologically, the FGAs have strong affinities for the dopamine type 2 (D2) receptor, whereas the SGAs are characterized by a more pronounced 5-HT2A antagonism than FGAs, and a lower potency for the D2 receptor (9, 10). The narrow focus on the dopaminergic system and striatal hyperdopaminergia in particular has most likely contributed to the limited evolvement of more effective drug treatment options as it has become increasingly clear that dopaminergic disturbances account for only parts of the clinical picture (particularly for positive symptoms) (11–13), and hyperdopaminergia is not present in all schizophrenia patients (14).

Recognizing the large impact that the schizophrenia associated cognitive dysfunctions have on functional outcome (15, 16), the efforts to improve the pharmacological treatment have moved toward cognitive enhancement, and pro-cognitive effects have been proposed as primary targets in future drug studies (17, 18). A major obstacle restricting hypothesis-driven pro-cognitive drug development has been the lacking knowledge of the structural and functional brain alterations underlying cognitive dysfunction in patients with schizophrenia (19, 20). Major scientific advances in unveiling the neurobiology of schizophrenia have emerged in recent years, however. The knowledge of transmitter dysfunctions especially is widened (21), accompanied by evidence of disturbances of myelination and inflammation/immunology in the pathophysiology of schizophrenia (22, 23). Thus, going beyond the neurotransmitter disturbances associated with schizophrenia, the present work reviews the latest evidence on myelin alterations, and the involvement of neuroinflammation/immunology in schizophrenia, with putative relevance for pro-cognitive drug effects in schizophrenia and related psychotic disorders. Moreover, these areas seem at least partly inter-related and the review aims at integrating the evidence from key publications in a joint model. Increased insight into the relevant functional and structural alterations might pave the way for more efficacious, targeted, and personalized pro-cognitive drug treatment opportunities aimed at improving real-world functioning. The review focuses on both previously unknown treatment targets for existing antipsychotic drugs, and potentially novel pro-cognitive drugs addressing extra-dopaminergic treatment targets, and gives an update also commenting on current constraints of pro-cognitive drug development for patients with schizophrenia.

## Cognitive Dysfunction in Patients with Schizophrenia

Cognitive function may be understood in terms of inter-related complex neural networks, influenced by different neurotransmitters, cytokines, and other substances including brain-derived neurotropic factor (BDNF) acting as neuromodulators (24).

Cognitive deficits are viewed as core characteristics of schizophrenia, with clinically significant cognitive impairments observed in a large majority of patients, and most patients experience lower cognitive functioning than would be expected from parental levels of education (16, 25). Cognitive deficits are seen before the development of psychosis (25–28), and cognitive decline and intellectual stagnation have been suggested to constitute a risk phenotype for developing schizophrenia (25). Furthermore, cognitive deficits are relatively stable after psychotic breakthrough and still present when the symptoms of psychosis have been treated (29, 30). A general cognitive dysfunction across cognitive domains is present, with additional selective deficits in working memory, executive function, attention, verbal fluency, episodic memory, and processing speed (15, 31). The effect size of the cognitive dysfunction compared to healthy subjects is close to 1.0 (32, 33), and in several neurocognitive dimensions the impairment is between 1 and 2 SDs, indicating a clinically significant loss of function (25, 34–37). Cognitive functioning has greater impact than positive psychotic symptoms in determining the patient’s real-world functional outcome (15, 38–41), and relationships between more specific cognitive domains and functional domains have also been reported (38, 40, 42). Thus, cognitive impairment has been put forward as the main target for novel treatment interventions in schizophrenia (25).

A significant challenge has been to develop clinical meaningful measures of cognitive dysfunction for pro-cognitive drug advancement. A major contribution to establish a standardized and manageable test battery for measuring cognition in schizophrenia has been the development of the Measurement and Treatment Research to Improve Cognition in Schizophrenia (MATRICS) Consensus Cognitive Battery (MCCB) (43). The MCCB composite score has been suggested as the best outcome measure based on findings of low practice effects, high test–retest reliability, and good external validity (44), while a recent factor analysis identified a three factor model of processing speed, attention/working memory, and learning as the best representations of data in this particular study (45).

## Does Abnormal Myelination Cause Cognitive Dysfunctions in Patients with Schizophrenia?

Myelin, the main constituent of white matter, is essential for controlling and regulating conduction velocities along axons and thus the synchronicity of brain signals across different brain regions, and for controlling synapse plasticity (46). Decreased myelin integrity might accordingly alter normal signal transduction. In the following, we will focus on one major line of recent achievements in schizophrenia research, namely the rapidly expanding knowledge of abnormal central nervous myelination in patients with schizophrenia and the possibilities to prevent or repair myelin pathology. White matter brain alterations and functional connectivity problems that have been linked to cognitive dysfunctions in schizophrenia will be briefly described, followed by in-depth descriptions of new findings of myelin alterations linked to cognition. Again, a major problem is to identify manageable tests/measures of myelinization and myelin integrity that can be used in drug discovery programs in a reliable way. Finally, we will discuss candidate drug treatments counteracting deficits of myelinization in patients with schizophrenia

### White matter changes and cognition

Several distinct alterations in gray and white matter are well established for schizophrenia patients as a group (47). Reviews of white matter tract pathology in patients with schizophrenia [mostly studies based on voxel-based morphometry (VBM) and diffusion tensor imaging (DTI)] have identified widespread and, in many studies, progressive changes (47). A meta-analysis of 15 DTI studies by Ellison-Wright and Bullmore (48) identified reductions (in all studies) of left frontal deep white matter and left temporal white matter. Thus, the authors suggested that two different networks may be affected; one connecting the frontal lobe with thalamus and cingulate gyrus, the other connecting the temporal lobe with, e.g., insula, amygdala, hippocampus, and the frontal lobe. A review by Walterfang et al. (49) of white matter pathology in patients with schizophrenia identified changes in the left uncinate fasciculi as the most replicated finding, furthermore that deficits of information processing was associated with volume reductions in the uncinate and inferior longitudinal fasciculi, anterior internal capsule, and corpus callosum (49). White matter pathology related to cognition has also been found in first-episode patients with psychosis. For example, Rigucci and colleagues (50) reported a relationship between speed of processing and visual memory and white matter fractional anisotropy (FA) in fronto-temporal tracts. Focusing on intra-cortical white matter, several clusters of superficial white matter (SWM) composed of U-shaped fibers connecting neighboring gyri together with intra-cortical axons and fibers from deep matter pathways displayed reduced FA in a study comparing patients with schizophrenia to healthy controls. The correlations between SWM-FA and cognition displayed in healthy controls were missing in patients (51).

### Functional connectivity studies

Recently, also related to myelination, the search for brain alterations underlying the psychopathology of schizophrenia has refocused to examine neuronal dysconnectivity, i.e., abnormal communication between local and distributed neuronal circuits or disrupted integration of brain activation (52–54). Fornito et al. (53) reviewed data on brain connectivity, and concluded that schizophrenia is associated with: (1) a widespread and possibly context-independent connectivity deficits, (2) additional transient states of hyper- and/or hypo-connectivity related to specific tasks.

Disturbed connectivity within the frontal lobes has been found to be related to working memory and to executive functioning (52, 54). This includes abnormal connectivity in fronto-parietal, fronto-cerebellar, and fronto-hippocampal networks (52). Verbal processing deficits have been shown to be related to circuits in the language areas of the brain, particularly to fronto-temporal dysfunction (52, 54). Indeed, theories have been developed concentrating on the effect of reduced executive cognitive control over the language processing in the temporal lobes as central to the development of the symptoms of psychosis, for instance auditory verbal hallucination (55).

Of future interest may be the influence of antipsychotic drugs on the dynamic interaction between large-scale cortical networks related to resting and effort-mode states (56). Using fMRI, Hugdahl et al. (57) found overlapping activation in a large-scale network to occur with a range of different cognitive tasks implicating the prefrontal cortex [see also Ref. (58)] suggesting that effective cognitive processing would require down-regulation of the resting-state network in situations with increasing demands for the up-regulation of the effort-mode network, and that cognitive impairment in schizophrenia may result from failure of network up- and down-regulation dynamics. Thus, based on brain imaging results, therapeutic agents inducing stabilization and, ideally, normalization of large-scale cortical network dynamics could possibly have pro-cognitive effects. However, thus far no drug has been reported to enhance connectivity (52).

The major question related to the structural basis of functional dysconnectivity was addressed in the review by Fornito et al. (53). The authors concluded with major overlaps between functional and structural findings, but also differences, which could at least partly be caused by intrinsic problems of the methods of structural measurements (see Measuring Myelination).

### Measuring myelination

Several methodological issues need consideration regarding the use of imaging techniques for myelin assessments. The DTI signal may reveal changes in white matter integrity but has limitations in identifying the exact nature of these changes, as the FA reflects different processes like demyelination, axonal swelling, or atrophy, and is sensitive to confounding effects of fiber crossings (59). In a recent review by Du and Öngür (60), two novel MR-based approaches – diffusion tensor spectroscopy (DTS) and magnetization transfer ratio (MTR) – were reviewed as measures of axonal diameter (DTS) and myelin volume (MTR). Combining MTR with DTI, Palaniyappan et al. (61) recently showed that the degree of FA reduction in areas with decreased myelin volume predicted impaired processing speed, leading the authors to suggest that combined DTI/MTR could be used as a treatment measure in the development of pro-cognitive drugs.

A study applying graph theory to analyze data from DTI combined with MTR showed that patients with schizophrenia have a weaker globally integrated structural brain network when compared to healthy controls (62). The same study concluded that the frontal hubs had a less central role than in healthy controls, which is in accordance with a reduced structural capacity to integrate information across brain regions. Moreover, a new study combining DTI and resting-state fMRI comparing patients with schizophrenia to healthy controls identified a selective disruption of brain connectivity among central hub regions with a potential communication capacity reduction and brain dynamics alterations (63).

### Myelin deficiencies in schizophrenia

Compelling evidence from several different lines of research has, in recent years, implicated dysfunctional myelin and oligodendrocytes, the cells responsible for wrapping myelin around neuronal axons in the brain, in the pathology of schizophrenia and related psychoses (22, 64–66). Sources of evidence include genetics and pharmacogenetic studies, post-mortem studies, and as briefly reviewed in the previous subsections of brain imaging studies, particularly DTI studies.

Normal myelination starts in the late part of fetal life and occurs most rapidly during the first 2 years after birth, but continues to adulthood in a region- and brain matter-specific manner (22, 65, 67). Initial myelination occurs subcortically, thus increasing the conduction velocities across different brain regions while leaving the intra-cortical portion of the axon unmyelinated during childhood (65). Interestingly, maturation of white matter in the prefrontal cortex and in fronto-temporal tracts occurs later, in late adolescence and early adult life, coinciding in time with the peak age of schizophrenia onset (1). Possibly, disturbances in the maturation of myelin around the end of the myelination stage could be one of the events contributing to the peak debut incidence of schizophrenia in this age group (66). The prefrontal cortex is one of the regions most robustly implicated in schizophrenia pathology, and working memory performance, which engages this region, has been found to be positively associated with the level of white matter maturation (67). Later intra-cortical myelination occurs for the most part in adulthood and is associated with the refinement of cognitive functions and brain plasticity (65). Importantly, myelination is a dynamic process, and oligodendrocyte progenitor cells continue to differentiate throughout life (68). In schizophrenia, intra-cortical myelin deficits seems to be more pronounced than subcortical deficits, which have been found to be more strongly associated with the duration of illness. Indeed, evidence suggests that myelin integrity is closely related to both cognitive functioning and to the symptoms of a variety of psychiatric and neurological disorders (22, 46).

Alterations particularly in dopaminergic transmission (69, 70), and also in the glutamatergic system, (11, 71) have been robustly implicated in the pathology of schizophrenia for several decades. Glutamatergic *N*-methyl-d-aspartate (NMDA) receptor antagonists, such as phencyclidine (PCP) and ketamine, have the potential to induce not only positive symptoms of psychosis but also negative symptoms and cognitive dysfunction resembling those seen in schizophrenia (11, 71). This has given rise to the NMDA receptor hypofunction hypothesis of schizophrenia (21, 72). The hypothesis has been extensively investigated in animal models but several limitations related to how well evidence from preclinical studies translate into schizophrenia patients have been pointed to (72). Seemingly, paradoxically another model of schizophrenia-related cognitive dysfunction is that of glutamatergic hyperactivity (73). The model is based on other observations of increased prefrontal glutamate release following NMDA receptor antagonism. The apparent paradoxical glutamate findings might be explained by different NMDA receptor properties related to the different locations and/or sub-compositions of the different NMDA receptors as elaborated by Zhou et al. (74). Interestingly, there are several points of convergence between myelin deficits and neurotransmitter alterations. Firstly, glutamate is excitotoxic when in excess, and oligodendrocyte progenitor cells display great vulnerability for glutamatergic excitotoxicity (68). Increased glutamate levels have been found in some phases of schizophrenia (75–77). Secondly, there is emerging evidence that dysfunctional myelin and oligodendrocytes could increase striatal dopamine levels, see Ref. (22) for details. One example could be that if glutamatergic projections from the prefrontal cortex to brainstem areas suffer from myelin damage, this might lead to lower excitatory input at inhibitory brainstem GABAergic interneurons, resulting in less inhibition of dopaminergic mesolimbic projections and consequently striatal hyperdopaminergia as outlined by Schwartz et al. (78).

### Myelin as drug target for present antipsychotics

Animal studies suggest that myelin-protecting and oligodendrocyte-stimulating properties may be a therapeutically relevant mechanism of action for at least some SGAs (61, 79, 80). In a DTI study of schizophrenia patients in exacerbation, Garver et al. (81) found increased diffusibility consistent with decreased myelin integrity during acute psychosis. After initial examination, patients were treated with the SGAs risperidone or ziprasidone, or the FGA haloperidol, with partial restoration of myelin integrity after 4 weeks in the subgroup that responded to treatment. Bartzokis et al. found higher volumes of intra-cortical myelin in schizophrenia patients treated with oral risperidone compared to those treated with the FGA fluphenazine decanoate (82). In a recent study by the same group, the long acting injection formulation of risperidone was associated with increased intra-cortical myelin volume compared to the oral formulation of the same compound (64). Myelin primarily consists of lipids, particularly cholesterol, which are mainly synthesized *de novo* in the brain. Interestingly, several antipsychotic agents have been demonstrated to directly induce lipogenesis through the sterol regulatory element binding protein (SREBP) system, and these lipogenic effects have been suggested to contribute to myelin-stimulating effects of antipsychotic agents (83–85). In this regard, it is highly interesting that clozapine, with its superior clinical efficacy, is also among the antipsychotics associated with the most pronounced metabolic adverse effects; in fact, a correlation between clinical improvement and increase in serum lipid levels has repeatedly been demonstrated (86–88). Summing up, a small but consistent body of evidence indicates that some current SGAs have positive effects on myelin volume, with possible distinctions among drugs and drug formulations.

### Other potential “myelin-enhancing” treatment options

In a clinical randomized controlled trial (RCT) by Amminger et al. (89), a markedly decreased progression rate to psychosis was found in at risk subjects receiving high-dose polyunsaturated fatty acids (PUFAs). PUFAs are involved in the myelination process, and peripheral PUFA levels have been found to be decreased in schizophrenia (90, 91). A recent DTI study in early-phase psychosis patients found an association between level of PUFAs in peripheral erythrocytes and white matter integrity (90). Possibly, PUFA distribution is altered in patients at risk for psychosis, with a link between PUFA levels and white matter integrity. Free radicals can damage membrane PUFAs, and disturbances in fatty acids and membrane phospholipid identified in patients with schizophrenia may be caused by increased oxidative stress according to a review by Yao and Keshavan (92). The same authors point to disruption of antioxidative systems related to schizophrenia, with reduced amounts of non-enzymatic plasma antioxidant components [e.g., albumin, bilirubin, uric acid, ascorbic acid (vitamin C), α-tocopherol (vitamin E)], see also the recent clinical study by Zhang et al. demonstrating a reduced plasma total antioxidant status in a sample of schizophrenia patients (93). Interestingly, PUFAs also have mild anti-inflammatory effects, see Section “Additional Drugs with Anti-Inflammatory Action as Add-on Treatments for Patients with Schizophrenia.”

## Inflammation and Immunology in Schizophrenia

### Implicating inflammatory systems in schizophrenia

Several findings point to a link between inflammatory processes and the pathophysiology of schizophrenia: (1) activated peripheral inflammatory system and neuroinflammation in patients with schizophrenia (94, 95), (2) evidence from genetic studies of correlation between schizophrenia and genes encoding for different components of the immune system (96–98), (3) post-mortem studies demonstrating up-regulated immune genes in the prefrontal cortex of patients with schizophrenia (99), (4) findings that the raised risk of schizophrenia seen after maternal infections acts via immunological mechanism (23), and (5) psychotic symptoms and cognitive dysfunction caused by immunological neurological syndromes (100), e.g., the interesting line of pathophysiological evidence based on findings in autoimmune synaptic encephalitis (limbic encephalitis), where antibody formation against NMDA receptors is associated with a wide range of psychiatric symptoms, in some patients also with syndromes resembling schizophrenia (100). Binding of NMDA antibodies has been found predominantly in the hippocampus (101). Also, in systemic autoimmune disorders such as systemic lupus erythematosus (SLE), patients can suffer from various psychiatric syndromes, but the pathophysiology has yet to be characterized in detail (102). A recent remarkable finding is that the levels of soluble receptors for the pro-inflammatory cytokine tumor necrosis factor (TNF)-α correlates with function in patients with schizophrenia compared to healthy individuals (103), the same study also found increased levels of TNF-α in the treatment resistant group compared to treatment responders.

In the following sections, we highlight some of the main findings related to inflammation/immunology and schizophrenia, with the specific purpose of evaluating the status of immune-modulating treatment with cognitive-enhancing effects.

### Cytokines

In the context of inflammatory changes in schizophrenia, the majority of findings stem from the innate system, but components of the adaptive immunology system have also been implicated. The macrophages of the innate system induce the release of acute-phase proteins [e.g., C-reactive protein (CRP) from hepatocytes] as well as producing and releasing cytokines (97), all markers of inflammation (104). Cytokines are also produced by peripherally activated endothelial cells and monocyte-derived dendritic cells (97).

### Peripheral inflammatory findings in schizophrenia

Multiple studies have shown low-grade disturbances in the peripheral inflammatory system in patients with schizophrenia, see recent reviews (105–107). Pro-inflammatory changes include elevated serum measures of pro-inflammatory cytokines [interleukin (IL)-1β, IL-6, IL-8, TNF-α] and other pro-inflammatory factors [prostaglandin E_2_ (PGE_2_), CRP] and elevated monocyte counts and activated immune cells have been associated with schizophrenia (108). Interestingly, some cytokines [IL-12, IFN-γ, TNF-α, soluble IL-2 receptor (sIL-2R)] may be trait markers for schizophrenia, while others are raised during phases of intensified symptoms (IL-1β, IL-6, and TGF-β) (106, 108). Furthermore, some anti-inflammatory substances are elevated in subgroups of patients with schizophrenia [soluble IL-1 receptor antagonist (sIL-1RA), soluble IL-2 receptor antagonist (sIL-2RA), soluble TNF receptors (sTNFRs)1, sTNFR2 (109), IL-10 (a cytokine with anti-inflammatory properties), and transforming growth factor (TGF)-β] (105). A review (110) identified decreased levels of IL-2 in patients with schizophrenia. IL-2 serves important functions in T-cell-mediated immunologic reactions. Although the findings of disturbances in the peripheral inflammatory system are not without inconsistencies, and the measurements of cytokines are sensible to a wide range of confounders (e.g., the influence of antipsychotic medication and metabolic status), there is firm evidence for a mixed picture of peripheral pro- and anti-inflammatory changes in a large proportion of patients with schizophrenia.

### Central inflammatory findings in patients with schizophrenia

Brain neuroinflammation, in contrast to inflammation in other parts of the body, does not lead to leukocyte recruitment, but is characterized by activated microglia, the mononuclear phagocytes of the brain (111). Microglia develop from primitive myeloid progenitors, and have various roles in the developing human brain including synaptic pruning, remodeling of axons, neuronal differentiation, and programed cell death (112). PET studies using (R)[11C]PK11195 (95, 113) have found microglial activation in schizophrenia, in addition to post-mortem studies demonstrating increased density and activation of microglia, though some of these studies have yielded conflicting evidence (112). Activated microglia have increased levels of the translocator protein (TPSO) located at the mitochondrial membrane, and the binding of TPSO to the (R)[11C]PK11195 is utilized in PET studies as a measure of neuroinflammation, while other PET ligands for activated microglia are under development (113). Free-water imaging (114) is another mode of neuroinflammation imaging, and studies using this novel method have indicated widespread brain inflammation and early signs of neurodegeneration in first-episode patients with schizophrenia (115).

#### Microglia, astrocytes, and the kynurenine pathway

**Figure 1:**
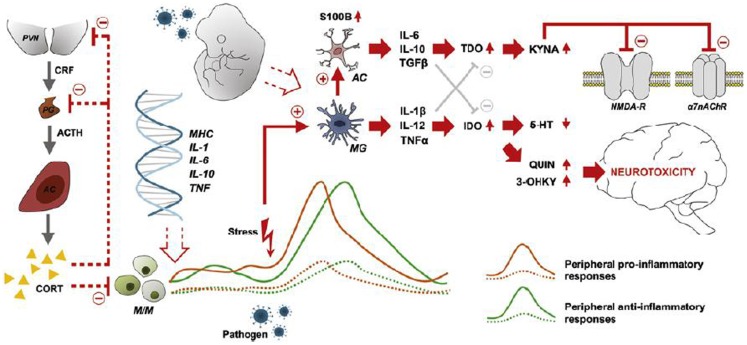
**Characteristics, sources, and neuronal consequences of activated inflammatory responses in schizophrenia**. Abnormal expression of inflammatory genes in monocytes/macrophages (M/M) results in peripheral low-grade inflammation (patients: solid orange and green lines-healthy subjects: dashed lines). Stress and/or pathogen exposure may lead to an over-activation of microglia (MG), which activates astrocytes (AC) and the consequent release of cytokines (IL-6, IL-10, TGF-β) that stimulate the production of kynurenic acid (KYNA). KYNA blocks signaling at the NMDA receptor and the α7 nicotinic acetylcholine receptor (α7nAChR). MGs releases cytokines (IL-1β, IL-12, TNF-α) that weaken the biosynthesis of serotonin and promotes the production of quinolinic acid (QUIN) and 3-hydroxykynurenine (3-OHKY), both neurotoxic substances. Abnormal HPA axis function may further contribute to the development of low-grade inflammation as the normal feed-back exerted by cortisol is not working properly. Furthermore, prenatal immune priming by *in utero* exposure to infection may provide a developmental source of long-term immune abnormalities to schizophrenia. With permission from Meyer et al. (84).

Activated microglia have many important functions besides phagocytosis, including cytokine production (IL-1β, IL-12, and TNF-α) (94, 111), most importantly crosstalk with the serotonin and glutamate neurotransmitter systems, involving the kynurenine pathway (116). Activated microglia also induce functional changes in brain astrocytes, which release IL-6, IL-10, and TGF-β (94). In the presence of pro-inflammatory cytokines, e.g., IFN-γ and TNF-α, tryptophan catabolism to kynurenins in microglia and astrocytes is induced, resulting in the production of quinolinic acid (QUIN) in microglia and KYNA in astrocytes. QUIN is an NMDA agonist and potentially excitotoxic, while KYNA is an NMDA antagonist and also blocks the α7 nicotinic acetylcholine receptor (α7nAChR) (94, 116). Figure 1 summarizes major points of the activated inflammatory responses in schizophrenia. The demonstration of elevated levels of KYNA in the cerebrospinal fluid (CSF) of patients with schizophrenia by two independent groups (117, 118) in 2001 boosted research in brain inflammatory-related processes implicated in schizophrenia. Elevation of KYNA in the CSF and prefrontal cortex in patients with schizophrenia is now an established finding, and is probably a consequence of peripheral inflammation according to a recent review by Schwarcz et al. (119). Elevated levels of KYNA are associated with decreased cognitive performance in animal models, including altered pre-pulse inhibition of the auditory startle response, deficits of working memory, and attentional set-shifting paradigms (120, 121), while reducing KYNA resulted in improved cognitive performance in a wide range of tasks (121). Adding to this, the kynurenic pathway also offers a possible explanation of how stress induces psychotic symptoms and cognitive dysfunction in schizophrenia by linking increased levels of glucocorticoids to the production of kynurenine in liver, subsequently converted to KYNA in the brain (121). The kynurenic pathway offers several potential drug targets for the reduction of brain KYNA levels, which could improve cognitive function in schizophrenia. Candidate pharmacological agents related to schizophrenia treatment block, e.g., the enzyme kynurenine aminotransferase (KAT), which transforms kynurenine into KYNA. Most KYNA-lowering drugs still lack behavioral data (122), while cerebrolysin, a peptidergic drug manufactured from purified porcine brain preparations, which lower brain tissue KYNA levels *in vitro* (123) also have been found to improve cognition (124). Interestingly, in a double blind RCT of patients with schizophrenia cerebrolysin (intravenous infusion) as add-on treatment to risperidone was found to improve scores at the Wechsler Adult Intelligence Scale and the Wechsler Memory Scale (124). Cerebrolysin is generally well-tolerated (125) and has also demonstrated pro-cognitive effects in patients with dementia (126, 127) and traumatic brain injury (128). In another recent study, d-cycloserine (DSC), a partial agonist at the glycine site of the NMDA receptor, was also found to reduce the levels of KYNA in human post-mortem frontal cortex homogenates by dose-dependently blocking KAT (129). DSC has also been found to improve memory consolidation and to reduce delusions in combination with cognitive behavioral therapy (130).

#### IL-1β, S100B, BDNF

In addition to elevated KYNA/QUIN, other aspects of an activated brain immune system can also have clinically important effects in schizophrenia. The proinflammatory cytokine IL-1β has been linked to glutamatergic excitotoxicity (131), and close dynamic interactions between IL-1β and the NMDA receptor was the recently demonstrated (132). Pro-inflammatory cytokines (e.g., IL-1) are also candidate causal agents implicated in the cerebral myelin damages discussed above, see (133, 134). The protein S100B, produced by astrocytes, is a marker of astrocyte activation. Serving as an example of the pleiotropic effects associated with many inflammatory factors at low concentration, S100B has beneficial effects, limiting microglia activation, stress-related neuronal damage, and increase glutamate uptake by astrocytes, while at higher concentration it has detrimental effects, including induction of microglial TNF-α secretion and induced production of, e.g., COX2/PGE-2, IL-1β, etc. (135). A meta-analysis concluded that serum levels of S100B may be elevated in schizophrenia, and disruptions of the blood–brain barrier (BBB) and/or active secretion by astrocytes were suggested as possible causes (136).

The neurotrophin BDNF is among the substances secreted by immune cells during inflammation and immune responses (137), and an inverse relationship between levels of BDNF and certain cytokines has been found (138). BDNF has been implicated in widespread processes relevant to the pathophysiology of schizophrenia including neurodevelopment, synapse regulation, and neuroplasticity, as well as effects on cognition, see for example (138–140). BDNF has been found to be reduced in schizophrenia in both drug-naïve and chronic patients, although findings are not entirely consistent across studies. Furthermore, an association has been found between reduced neurocognitive function and reduced serum BDNF levels (139). BDNF interacts with dopaminergic, glutamatergic, and serotonergic systems in the brain, see Nurjono et al. (138) for a recent update. Regarding BDNF as a drug target, FGAs have been reported to lower peripheral BDNF, whereas SGAs either have no effect or increase BDNF, while antidepressants have been shown to increase BDNF (140).

### Structural and functional correlates of inflammatory disturbances in patients with schizophrenia

Structural brain alterations associated with immune-related genetic polymorphisms were recently reviewed by Fineberg and Ellman (107). The review identified associations between variability in the IL-1 gene complex and white and gray matter volume reductions in temporal and frontal brain regions, together with enlarged ventricles in patients with schizophrenia. Furthermore, this review discussed associations of IL-1 gene variations with decreased dorsolateral prefrontal function during different neurocognitive tasks (107). In another review, Meyer (105) found evidence for a positive correlation between the severity of cognitive deficits and the peripheral levels of IL-1β, IL-6, TNF-α, CRP, and S100B. While the correlation of the inflammatory disturbances with specific cognitive effects is not clarified in patients with schizophrenia, findings from animal studies and studies in non-schizophrenic individuals reveal associations of inflammatory disturbances with executive function, sustained attention, and working memory according to the review by Meyer et al. (105).

### Anti-inflammatory effects of antipsychotic drugs

Monji et al. (141) recently reviewed studies (mostly *in vitro* and animal studies) concerning the capability of antipsychotic drugs to influence activated microglia. The review identified several studies showing that antipsychotic drugs significantly reduce the secretion of TNF-α, nitric oxide, IL-1β, and IL-2 from activated microglia. Furthermore, some drugs were found to have stronger inhibitory effects, e.g., risperidone, which inhibited the secretion of several cytokines from activated microglia more than haloperidol, and indications of clozapine-specific effects have also been reported. Moreover, in the context of the kynurenine pathway, one animal study demonstrated that chronic treatment with antipsychotic drugs reduces brain KYNA (142). Meyer et al. (94) reviewed the influence of antipsychotic treatment on peripheral cytokines in clinical populations and found that antipsychotics reduced the level of proinflammatory cytokines (IL-1β, IL-6, sIL-6R, TNF-α) while increasing peripheral production of anti-inflammatory substances such as (sIL-1RA, sIL-2R and IL-10). Here, SGAs seemed to have the most pronounced effects and a possible relation between the ability to normalize immune function and clinical effects were identified. Brain imaging studies investigating antipsychotic drug treatment in brain imaging paradigms capable of showing effects on neuroinflammation (e.g., PET/“free water” MRI) would truly improve this field of research, but we have not been able to identify any such studies.

### The effects of anti-inflammatory/immune-modulating drugs in the treatment of schizophrenia

#### Non-steroid anti-inflammatory drugs

Based on the notion of activated immune responses in the brain and/or peripheral tissues, a few clinical studies have evaluated the effects of non-steroid anti-inflammatory drugs (NSAIDs) in schizophrenia. The pooled effects size in reducing total Positive and Negative Syndrome Scale (PANSS) score according to a recent meta-analysis of the effect of add-on treatment with NSAIDs [eight studies, two with aspirin (*n* = 270), six with celecoxib (*n* = 504)] to different antipsychotics showed non-significant benefit over placebo (143). Treatment with aspirin alone was superior to placebo, while treatment with celecoxib was not; these conclusions were also drawn in a meta-analysis of Sommer et al. (144). When the studies were categorized according to disease phase, the pooled analyses of all eight studies revealed positive effects of NSAIDs vs. placebo for first-episode patients. The authors concluded that the present data do not lend significant support to NSAIDs as add-on therapy to antipsychotic treatment for patients with schizophrenia. Another review agreed that indiscriminately suppressing inflammation seems not to be the optimal way of treating immunological disturbances associated with schizophrenia, and that treatment should aim at reversing glial loss, upregulating beneficial microglial activation and proliferation (MAP) together with other neuroprotective measures, while ideally down-regulating harmful aspects of MAP (135). At present, studies more precisely aiming to elucidate the putative pro-cognitive effects of treatment with NSAIDs in patients with schizophrenia are lacking, although non-significant effects were demonstrated in a small study (145).

#### Erythropoietin

Erythropoietin (EPO) is a cytokine with several identified functions in the brain (146). In a 12-week, placebo-controlled study (*n* = 39) with stable schizophrenia patients, treatment with recombinant human EPO (40000 IU/week) improved neurocognitive function compared to placebo in the domains of delayed memory, language-semantic fluency, attention and perseverative errors (147). An evaluation of gray matter development in the same study showed that gray matter loss was significantly less pronounced in the group treated with EPO (148). Animal studies have supported a pro-cognitive effect of EPO treatment (146). A systematic review identified multiple putative beneficial brain targets of EPO treatment, e.g., modulation of inflammation, neuroprotection, neurotransmission regulation, effects on BBB permeability, but the use of EPO can be limited by adverse effects such as thrombosis and cancer (149). EPO has also been found to improve cognitive function in studies of patients with Parkinson’s disease (150) and multiple sclerosis (151).

#### Minocycline

The tetracycline antibiotic drug minocycline has also been tested as an add-on treatment for patients with schizophrenia, based on evidence from animal studies that showed minocycline to have multiple actions including inhibition of microglial activation and the attenuation of adaptive immunity through the reduction of activity and expression of matrix metalloproteinases (MMPs). MMPs enable T-cells to migrate through the BBB by altering its permeability, a process associated with myelin degradation (152). In a placebo-controlled, 6-month trial in patients with schizophrenia (54 patients receiving 200 mg minocycline and 26 patients receiving placebo), cognitive function (and negative symptoms) improved significantly in the minocycline-treated group, particularly in the domains of working memory, cognitive flexibility, and planning (153). Chaudry et al. (154) reported the results of a 1-year RCT of 155 early schizophrenia patients performed in Brazil and Pakistan, where minocycline reduced negative symptoms (adjusted difference 95% confidence interval 1.55–5.51 at the PANSS negative subscale), but no significant effects of treatment were found in tests for cognitive function. The meta-analysis of Sommer et al. (144) identified four RCT studies with *n* = 182 patients given minocycline, and *n* = 166 on placebo, the effects on cognition were not reported, but no significant effects on overall symptom severity were identified.

#### Additional drugs with anti-inflammatory action as add-on treatments for patients with schizophrenia

The meta-analysis by Sommer et al. (144) also investigated the effect of additional anti-inflammatory treatments. The outcome measure was the mean change in PANSS (155) or the Brief Psychiatric Rating Scale (156), and significant improvements of treatment with *n*-acetylcysteine (*n* = 140), aspirin (*n* = 270), and estrogens (*n* = 262) were identified, while studies with davunetide and PUFAs (eicosapentanoic acid and docosahexanoic acids) did not show significant effects.

### Summing up pro-cognitive effects of immunomodulating/anti-inflammatory drug treatment for patients with schizophrenia: Critical methodological problems remain

The involvement of inflammatory/immunological factors in the pathophysiology of a large portion of patients with schizophrenia is well established through different lines of research, and data point to several links between these factors and cognitive dysfunction. Presently, many drugs with anti-inflammatory or immunomodulatory action have been tested (as add-on to antipsychotic treatment) for patients with schizophrenia, and while some have shown positive effects for global schizophrenia symptoms, the effects on cognition were not possible to assess in the recent meta-analysis by Sommer et al. (144), as only 5 of 26 included studies reported data on cognitive tests and the heterogeneity of the cognitive tasks made it impossible to quantitatively review the effects. Furthermore, as highlighted by Sommer et al. the drugs tested that were shown to improve the symptoms of schizophrenia (*N*-acetylcysteine, estrogens, aspirin) have broad mechanisms of action, and it is not at all demonstrated that the improved symptoms are caused by anti-inflammatory actions. A further critical question to address in future drug research will be to establish reliable brain imaging methods to quantitatively assess neuroinflammation, as the levels of peripheral cytokines are only an indirect measure of brain neuroinflammation. PET studies with new ligands that specifically target activated microglia and the “free-water” MRI scanning seem promising, but extensive development remains before these methods are ready as outcome measures in drug discovery studies.

## Summary and Convergence

**Figure 2:**
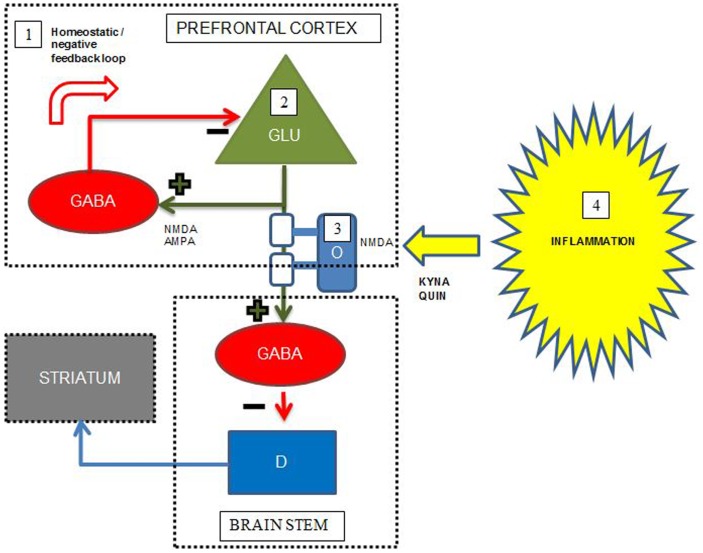
**Simplified putative interplay between selected neurotransmitters, oligodendrocytes, and inflammation in the pathology of cognitive dysfunctions in schizophrenia**. Green cell = excitatory pyramidal glutamatergic (GLU) neuron; red cell = inhibitory GABAergic (GABA) interneuron; blue cell = modulatory dopaminergic (D) neuron; light blue cell = oligodendrocyte (O) with white myelin sheets enclosing the pyramidal cell axon; KYNA = kynurenic acid, QUIN = quinolinic acid. The figure displays some putative points of convergence between neurotransmitters, myelin, and neuroinflammation relevant to the cognitive dysfunctions of schizophrenia. Details are elaborated in the text. (1) In the prefrontal cortex, abnormal NMDA functioning on, and/or deficient GABA release and reuptake in a subset of GABAergic interneurons result in deficient negative feedback on pyramidal glutamatergic neurons, which may lead to glutamatergic hyperactivity, excitotoxicity, and disruption of coordinated firing of pyramidal cells. (2) Deficient glutamatergic neuronal activity leads to decreased excitatory input on GABAergic interneurons terminating on dopaminergic projections to striatum, leading to striatal hyperdopaminergia. The hyperdopaminergia may inversely influence cognitive functions located in the prefrontal cortex. (3) Reduced myelin integrity may inversely influence glutamatergic signaling leading to the same consequences as in the point above. Oligodendrocytes express NMDA receptors and are thus vulnerable for excitotoxicity from excess glutamate and QUIN. (4) During inflammation QUIN is produced in microglia and KYNA in astrocytes. QUIN is an NMDA agonist and potentially excitotoxic, while KYNA is an NMDA antagonist that could induce cognitive dysfunctions consistent with the NMDA receptor hypofunction hypothesis of schizophrenia. Adapted with permission from Lisman et al. (72) and Takahashi et al. (102).

At present, pharmacological treatment of schizophrenia relies on antipsychotic drugs, which predominantly relieve positive psychotic symptoms and have little effect on the cognitive dysfunctions restricting the functional level of patients. However, recent developments in brain imaging, including novel specific PET ligands and MRI paradigms specifically investigating myelin integrity, neurotransmitter levels, and neuroinflammation, facilitate the unraveling of mechanisms underlying cognitive dysfunction in schizophrenia. Accumulating evidence supports a combined model including all these areas of pathology underlying the cognitive deficits in schizophrenia, schematically exemplified by Figure 2 for the interplay between prefrontal cortex, striatum, and brain stem. This joint model represents several potential pro-cognitive drug targets at the level of neurotransmitters, lipids, and inflammatory markers, however, no current drugs aimed at these targets are so far definitely proven to be clinically useful as pro-cognitive drugs for patients with schizophrenia. Studies concerning immunomodulatory drugs, including EPO, minocycline, celecoxib, aspirin, estrogens, *n*-acetylcysteine have shown promising results in single studies, but have not yielded straightforward support for the use in clinical practice to enhance cognition.

## Future Perspectives

Drugs that would reduce brain levels of KYNA (e.g., KAT inhibitors) are expected to improve cognitive dysfunctions in schizophrenia based on animal studies, but remain to be developed for clinical use. The possibility of pro-cognitive effects of drugs that have been used to treat other medical conditions for years, e.g., EPO, minocycline, and aspirin is attractive, also because the safety profile of many of these drugs are well known in neurological patients populations, reducing the costs of introducing the drugs as cognitive enhancers for schizophrenia patients. Several critical questions currently constraining progress remain to be solved, however. Firstly, uniformly accepted and standardized cognitive assessments for repeated measures must be agreed on for pro-cognitive drug studies in schizophrenia patients. Secondly, the refinement of brain imaging methods unto sufficient reliability and specificity (e.g., measuring neuroinflammation and myelin integrity) for use as outcome measures in pro-cognitive drug is essential for studies of putative cognition enhancers in humans. Thirdly, and at least of prominent importance in the field of further developments of pro-cognitive glutamatergic substances are the problems of translating findings in animal studies to human drug treatment.

However, even though the process of developing novel pharmacological agents is highly sophisticated, identifying novel treatment targets through thorough clinical and preclinical studies remains pivotal in the continued search for improved treatment of schizophrenia. The convergence of a disordered inflammatory system, disturbances in myelination/oligodendrocyte function, and transmitter dysfunctions in schizophrenia open new paths for pro-cognitive drug discovery with putative large functional improvements to be achieved for patients with schizophrenia.

## Conflict of Interest Statement

The authors declare that the research was conducted in the absence of any commercial or financial relationships that could be construed as a potential conflict of interest.
